# Sero-biochemical Studies in Sheep Fed with Bt Cotton Plants

**DOI:** 10.4103/0971-6580.72680

**Published:** 2010

**Authors:** B. Anilkumar, A. Gopala Reddy, B. Kalakumar, M. Usha Rani, Y. Anjaneyulu, T. Raghunandan, Y. Ramana Reddy, K. Jyothi, K. S. Gopi

**Affiliations:** Department of Pharmacology and Toxicology, College of Veterinary Science, Hyderabad, Andhra Pradesh, India; 1 Department of Veterinary Pathology, College of Veterinary Science, Hyderabad, Andhra Pradesh, India; 2 Livestock Research Institute, Hyderabad, Andhra Pradesh, India; 3 Department of Animal Nutrition, College of Veterinary Science, Hyderabad, Andhra Pradesh, India

**Keywords:** Bt cotton, sero-biochemistry, sheep

## Abstract

An experimental study was conducted to evaluate the toxicological effects, if any, due to feeding of Bt (*Bacillus thuringiensis*) cotton plants to sheep. A total of 32 sheep of one year of age belonging to *Deccani* breed were randomly divided into four groups, consisting of eight sheep in each group. Group 1 was maintained on basal diet (concentrate feed at the rate of 300 g + green fodder at the rate of 3 kg/sheep/day), group 2 on non-Bt cotton plant at the rate of 1.5 kg + green fodder at the rate of 1.5 kg + concentrate feed at the rate of 300 g/sheep/day, group 3 on Bt cotton plants (50%) at the rate of 1.5 kg + green fodder at the rate of 1.5 + concentrate feed at the rate of 300 g/sheep/day, and group 4 on Bt cotton plants *ad libitum* + concentrate feed at the rate of 300 g/sheep/day. All the groups of sheep were maintained for three months and various hemato-biochemical parameters were studied at monthly intervals. The activity of aspartate transaminase, *gamma* glutamyltransferase, and creatine kinase in sera samples, and the concentration of blood urea nitrogen and creatinine did not differ significantly among different groups at different time intervals. The histological examination of liver and kidney did not reveal any significant changes in Bt and non-Bt cotton-fed groups. In conclusion, the results of the present investigation enunciated that feeding of genetically modified (Bt) cotton plants to sheep was without detrimental effects in the biological system of sheep.

## INTRODUCTION

By introducing the genetic information encoding the insecticidal protein of Bt (*Bacillus thuringiensis*) into plants, the plants would produce their own insecticide (bio-insecticides) that reduces the application of pesticides and reduces environmental problems. Due to decline of grazing land, sheep and goats are let loose in the cotton fields for grazing by the farmers and shepherds after harvesting the cotton. The utilization of cotton plants and its by-products is limited due to the presence of gossypol in various parts of plants. Gossypol is a toxic component that is produced for defense by the plant and causes deleterious effects to nonruminants, although ruminants showed tolerance to gossypol toxicity.[1] Several studies showed that the amount of expression of Bt protein in plant is in very minute quantity and its action is limited to certain larval species and may not cause toxicity to mammals.[2] There were reports in the print media that sheep died in *Telangana* region of Andhra Pradesh after grazing on leaves and pods of harvested Bt cotton plant residue in fields.[3] Cultivation of Bt cotton has raised fears among the shepherds and other livestock owners over the safety of Bt cotton plants and their by-products. Therefore, a toxicological study was carried out to evaluate lipid profile, protein profile, and biomarkers of organ damage in sheep by feeding Bt cotton plants.

## MATERIALS AND METHODS

A total of 32 *Deccani* sheep of one year of age were randomly divided into four groups, consisting of eight sheep in each group, after an acclimatization period of two weeks. All the sheeps were dewormed and vaccinated, and provided with feed and water *ad libitum* throughout the experiment. Bt and non-Bt cotton plant crop was cultivated at Student Farm, College of Agriculture, Acharya N.G Ranga Agricultural University (ANGRAU), Hyderabad. All the groups were maintained as per the following treatment schedule for 90 days: Group 1, Basal diet (concentrate feed at the rate of 300 g + green fodder at the rate of 3 kg/sheep /day); Group 2, Non-Bt cotton plant at the rate of 1.5 kg + green fodder at the rate of 1.5 kg + concentrate feed at the rate of 300 g/sheep /day; Group 3, Bt cotton plants (50%) at the rate of 1.5 kg + green fodder at the rate of 1.5 + concentrate feed at the rate of 300 g/sheep/day; Group 4, Bt cotton plants *ad libitum* + concentrate feed at the rate of 300 g/sheep.

The blood samples were drawn from jugular vein on 30^th^, 60^th^, and 90^th^ day from the sheep in each group for estimation of aspartate transaminase (AST), *gamma* glutamyltransferase (GGT), creatine kinase (CK), blood urea nitrogen (BUN), and creatinine by using commercially available diagnostic kits (Qualigens Pvt. Ltd., Mumbai). Histological examination of liver and kidney was conducted to draw possible conclusions. The data were subjected to statistical analysis by applying one-way ANOVA using statistical package for social sciences (SPSS) 10^th^ version. Differences between means tested using Duncan’s multiple comparison test and significance was set at *P*<0.05.

## RESULTS AND DISCUSSION

The activity of AST (U/L) in the basal diet control was 34.50 ± 5.34, which was not significantly different when compared with the groups fed on Bt cotton, non-Bt cotton, and 50% Bt cotton (39.51 ± 4.00, 42.66 ± 4.30, and 36.55 ± 5.63, respectively) at the end of first month. Similar trend was observed on subsequent recording at the end of second month. The activity of AST in basal diet control at the end of third month was 35.13 ± 3.31, which did not differ significantly when compared with groups 3 and 4 (32.05 ± 2.86 and 37.53 ± 3.34, respectively). GGT activity (U/L) in the basal diet control group 1 was 20.61 ± 1.23, which did not differ significantly when compared to groups maintained with Bt, 50% Bt, and non-Bt cotton (20.52 ± 3.49, 22.68 ± 3.91, and 28.67 ± 4.06, respectively) at the end of first month. Similar trend was observed at the end of second month, and also showed no significant difference in GGT activity when compared with mean values of first month. The mean GGT activity of groups 1, 2, 3, and 4 at the end of third month (17.50 ± 2.96, 21.11 ± 4.62, 17.28 ± 3.65, and 21.11 ± 3.35, respectively) revealed a slight decrease when compared with respective means of second month. However, no significant difference was observed. The activity of AST and GGT was determined to assess the degree of damage to the liver as the levels of certain enzymes like alanine trnsaminase (ALT), AST, GGT, etc. are elevated following hepatocellular injury.[4] In this study, the activity of ALT and GGT was not significantly different among the groups. These results are further substantiated from the histopathological studies of liver, which did not reveal major histological changes except mild infiltration of mononuclear cells around central vein in liver.

The activity of CK (U/L) in group 1 (basal diet) was 11.09 ± 0.95 and revealed no significant difference when compared with groups 2, 3, and 4 (11.34 ± 3.09, 8.76 ± 1.15, and 7.48 ± 1.28, respectively) at the end of first month. Similar trend was observed at the end of second month of experiment. CK activity at the end of third month in groups 1, 2, 3, and 4 was 15.50 ± 2.70, 13.85 ± 1.89, 11.34 ± 2.24, and 13.98 ± 2.02, respectively, and there was no significant difference in the activity of CK. The skeletal musculature contains the largest CK among all other tissues and because of this, the specificity of CK measurement to monitor muscle damage is very high.[5] In the present study, the activity of CK was statistically nonsignificant among the groups.

The concentration of BUN (mg/dl) in control group was 43.88 ± 3.12 and it did not differ significantly when compared with groups 2, 3, and 4 (39.01 ± 4.12, 39.07 ± 4.11, and 38.45 ± 2.79, respectively) at the end of first month. Similar trend was observed at the end of second and third month. At the end, the mean values of BUN concentration in groups 1, 2, 3, and 4 were comparable with the respective mean values of second month and it was not significant. The serum creatinine concentration (mg/dl) in the control group was 1.26 ± 0.46, which was not significantly different when compared with groups 2, 3, and 4 (1.19 ± 0.07, 1.27 ± 0.007, and 1.18 ± 0.08, respectively) at the end of first month. The groups maintained with Bt and 50% Bt cotton revealed no significant change in the concentration of creatinine (1.43 ± 0.14 and 1.30 ± 0.06, respectively) when compared with that of basal diet control and non-Bt cotton (1.22 ± 0.13 and 1.30 ± 0.06, respectively) at the end of third month. The concentrations of non-protein nitrogenous substances such as serum creatinine and BUN were quantified to assess the nephrotoxicity. An increase in BUN reflects an accelerated rate of protein catabolism. Plasma urea appears to be the single most useful variable for detection of pre-renal causes of renal failure.[4] In the present experiment, the serum creatinine and BUN levels revealed nonsignificant difference in toxic control group at the end of 30, 60, and 90 days as compared with the remaining groups. Also, the histological examination of kidney did not reveal any tissue-specific damage.

In conclusion, the study did not reveal significant alterations in various sero-biochemical markers for organ damage following feeding of genetically modified cotton to sheep.

## References

[1] Reiser R, Fu HC (1962). The mechanism of gossypol detoxification by ruminant animals. J Nutr.

[2] Betz FS, Hammond BG, Fuchs RL (2000). Safety and advantages of Bacillus thuringiensis-protected plants to control insect pests. Regul Toxicol Pharmacol.

[3] Anonymous (2007).

[4] Kaneko JJ, Harvey JW, Michael LB (1997). Clinical Biochemistry of Domestic Animals.

[5] Boyd JW (1976). Creatine phosphokinase in normal sheep and in sheep with nutritional muscular dystrophy. J Comp Pathol.

